# LAPTM4B promotes the progression of nasopharyngeal cancer

**DOI:** 10.17305/bjbms.2020.4738

**Published:** 2021-06

**Authors:** Qun Su, Hongtao Luo, Ming Zhang, Liying Gao, Fengju Zhao

**Affiliations:** Department of Radiotherapy, Gansu Provincial Cancer Hospital, Lanzhou, China

**Keywords:** NPC, LAPTM4B, prognosis, proliferation, migration, invasion

## Abstract

Lysosomal protein transmembrane 4 beta (LAPTM4B) is a protein that contains four transmembrane domains. The impact of LAPTM4B on the malignancy of nasopharyngeal carcinoma (NPC) remains unclear. In the present study, we aimed to investigate the role of LAPTM4B in NPC. NPC tissue samples were used to evaluate the expression of LAPTM4B and its relationship with patient prognosis. Furthermore, we inhibited the expression of LAPTM4B in NPC cell lines and examined the effects of LAPTM4B on NPC cell proliferation, migration, and invasion. We found that LAPTM4B protein was mainly localized in the cytoplasm and intracellular membranes of NPC cells. LAPTM4B protein was upregulated in NPC tissues and cell lines. High LAPTM4B expression was closely related to pathological subtypes and disease stages in NPC patients. NPC patients with high LAPTM4B expression had a worse prognosis. *LAPTM4B* knockdown inhibited the proliferation, migration, and invasion ability of NPC cells. LAPTM4B plays a cancer-promoting role in the progression of NPC and may be a potential target for NPC therapy.

## INTRODUCTION

Nasopharyngeal carcinoma (NPC) is a malignant tumor originating from nasopharyngeal mucosal epithelial cells, which usually occurs in the pharyngeal recess. Compared with other malignant solid tumors, NPC shows lower morbidity and mortality rates. According to global cancer statistics, approximately 130,000 new NPC cases occur each year worldwide, accounting for 0.7% of all cancers [1]. Significantly, NPC cases are not uniformly distributed around the globe; the NPC incidence is higher in the East and Southeast of Asia. Especially in the southern region of China, the incidence of NPC is quite high: three cases per 100,000 individuals [2]. Of note, approximately 70% of NPC patients are already in stage III or IV at the time of diagnosis [3]. Still, the continuous progress of clinical/scientific research led to the development of comprehensive radiotherapy-based anti-tumor treatment, which has greatly reduced the mortality of NPC patients. However, tumor recurrence and distant metastasis are still the main factors impacting the long-term survival of NPC patients [4]. Therefore, it is imperative to elucidate the molecular regulatory mechanisms of NPC metastasis and find effective therapeutic targets.

Similar to other malignant solid tumors, the development of NPC is a complex process involving activation of oncogenes and inactivation of tumor suppressor genes. Lysosomal protein transmembrane 4 beta (LAPTM4B) is a protein that contains four transmembrane domains and is highly conserved among vertebrates [5]. LAPTM4B is expressed to varying degrees in human normal tissues: a high expression is observed in the testis, heart, skeletal muscle and uterus, while in the liver and lung the expression levels are very low [6]. Consecutively, studies have confirmed that LAPTM4B is abnormally expressed in different malignant solid tumors and plays a role in cancer promotion. A study on liver cancer has shown that the PPRP motif in the N-terminal region of LAPTM4B plays a key role in promoting tumor cell proliferation, migration, and invasion [6]. In another study, it was demonstrated that LAPTM4B interacts with epidermal growth factor receptor (EGFR) and Beclin 1 thereby promoting autophagy, while LAPTM4B knockdown reduces NPC cell radioresistance by inhibiting autophagy [7]. In addition, LAPTM4B is considered to be associated with metastatic breast cancer [8]. However, the impact of LAPTM4B on NPC remains unclear. In the present study, we aimed to investigate the role and underlying mechanism of LAPTM4B in NPC.

## MATERIALS AND METHODS

### Tissue samples

Paraffin-embedded tumor tissue samples were collected from 126 NPC patients admitted to the Gansu Provincial Cancer Hospital from 2005 to 2010. All the patients were diagnosed pathologically and had complete clinicopathological data and follow-up information. The patients were not subjected to radiotherapy, chemotherapy, or immunotherapy prior to sample collection. Among the 126 NPC patients, 93 were male and 33 were female with the mean age of 53.3 ± 10.6 years. This study was conducted in accordance with the Declaration of Helsinki and was approved by the Ethics Committee of Gansu Provincial Cancer Hospital (No. 20150228-16). Written informed consent was obtained from all patients.

### Cell culture and transfection

NPC cell lines (CNE-1, CNE-2, 5-8F, and 6-10B) as well as the human immortalized nasopharyngeal epithelial cell line (NP69) were originally obtained from Sun Yat-Sen University Cancer Center (Guangzhou, China). NPC cell lines were maintained in RPMI 1640 medium (Invitrogen, Carlsbad, CA, USA) supplemented with 10% fetal bovine serum (Gibco, Grand Island, NY, USA). NP69 was cultured in keratinocyte/serum-free medium (Invitrogen) supplemented with bovine pituitary extract (BD Biosciences, San Jose, CA, USA). All media were supplemented with 1% streptomycin/penicillin and the cells were cultured at 37°C in a humidified incubator containing 5% CO_2_. The medium was changed daily.

To assess the functional role of LAPTM4B, we suppressed the endogenous expression of *LAPTM4B* using small interfering RNA (siRNA) technology. The siLAPTM4B siRNAs (siRNA1: 5¢-GCAAGCTACATCCTACTGCTT-3¢; siRNA2: 5¢-CCAAATCTGATGGACCTAGAA-3¢) were purchased from GenePharma (Shanghai, China). NPC cells not subjected to any treatment were used as the negative control (NC group). NPC cells were seeded at 2 × 10^6^ cells per well in 6-well plates. They were then transfected with siLAPTM4B using Lipofectamine^®^ 2000 reagent; and later, the interference efficiency was determined by Western blotting.

### Immunohistochemistry

Paraffin-embedded tissue sections were cut into 4 mm. The sections were dewaxed using xylene and hydrated in solutions with increasing ethanol concentrations. Citrate buffer was used for antigen retrieval. Then, hydrogen peroxide was used to eliminate endogenous peroxidase activity. Afterwards, anti-LAPTM4B antibody (1:100; Abcam, Cambridge, UK) was added to the tissue sections and incubated at 4°C overnight. The next day, the secondary antibody was added to the preparations and incubated for 1 hour at room temperature. The final steps were color development and counterstaining.

For the expression of LAPTM4B, the staining intensity and staining ratio of tumor cells was analyzed. The staining intensity was classified as follows: 1, weak; 2, moderate; and 3, strong. Tumor cell positive staining was classified: 0, <5%; 1, 5–24%; 2, 25–49%; 3, 50–74%; and 4, ≥75%. Immune response was calculated by multiplying these two indicators. When the final score was >3, LAPTM4B was considered as highly expressed.

### Western blotting

When the cells grew to about 90% confluence, the cells were lysed using RIPA lysis solution (Beyotime Biotechnology, Shanghai, China), collected with a 1.5 ml centrifuge tube, and processed using an ultrasonic disruptor. Protein concentrations were determined using a BCA protein quantification kit (Solarbio Science and Technology Co., Ltd., Beijing, China). Proteins were separated by 10% sodium dodecyl sulfate-polyacrylamide gel electrophoresis and then transferred to polyvinylidene difluoride membranes. Membranes were blocked with 5% skimmed dry milk for 1 hour at room temperature and subsequently incubated with anti-LAPTM4B antibody (1: 1500; Abcam) overnight at 4°C. Membranes were washed the next day using Tris-buffered saline, Tween 20 (TBST) followed by incubation with secondary antibody and subsequently washed with TBST again. The final detection was performed by exposing the membranes with an ECL chemiluminescent substrate kit (Thermo Fisher Scientific, Waltham, MA, USA). The bands were scanned by ImageJ and the gray values were determined for performing densitometry.

### Cell counting kit-8 (CCK-8) assay

The effect of LAPTM4B on NPC cell proliferation was examined using CCK-8 assay. Specifically, cells were seeded at 8 × 10^3^ cells per well in 96-well plates and incubated overnight. A 20 µl of CCK-8 reagent (Beyotime Biotechnology) was added to each well following the kit instructions. Finally, each well was detected spectrophotometrically at 450 nm using a microplate reader (BioTek Instruments, Inc., Winooski, VT, USA). The experiment consisted of three independent replicates and all results were averaged.

### Transwell migration and invasion assays

The effect of LAPTM4B on NPC cell migration and invasion was assessed by transwell assays. For migration experiments, 1 × 10^5^ cells were seeded and cultured in the upper chamber of a Transwell plate (Corning, Inc., Corning, NY, USA) with serum-free medium and medium containing 10% serum was added to the lower chamber. After 24 hours, residual cells were removed using a cotton swab and subsequently fixed with 4% paraformaldehyde for 30 minutes. Finally, crystal violet staining was performed, and six high-power fields were randomly selected for cell counting under the microscope (Olympus, Tokyo, Japan). For invasion assays, precast Matrigel (Corning) was previously added to the Transwell chambers. The rest of the protocol was the same as in the migration assays described before.

### Wound healing assay

The effect of LAPTM4B on NPC cell migration was examined using the wound healing assay. Cells were seeded in 6-well plates and when the plated cells reached about 80% confluence, the 6-well plates were uniformly scratched three times in each well using a 200 μl sterile pipette tip. The detached cells were subsequently rinsed with phosphate-buffered saline, the scratch healing at 0 hour and 24 hours at each site was observed and photographed using the microscope (Olympus) and subsequently used to calculate the corresponding healing area.

### Statistical analysis

All data are presented as mean ± standard deviation (SD). The Chi-square test was used to compare categorical data between the groups. The t-test was used to compare the measurement data between the groups. Survival curves were plotted against survival data using Kaplan–Meier plotter and compared by log-rank test. The primary outcome measure was overall survival (OS). All analyses were performed using GraphPad Prism version 7.00 for Windows (GraphPad Software, La Jolla California USA, www.graphpad.com). Statistical significance was considered when *p* < 0.05.

## RESULTS

### LAPTM4B is highly expressed in NPC

**FIGURE 1:**
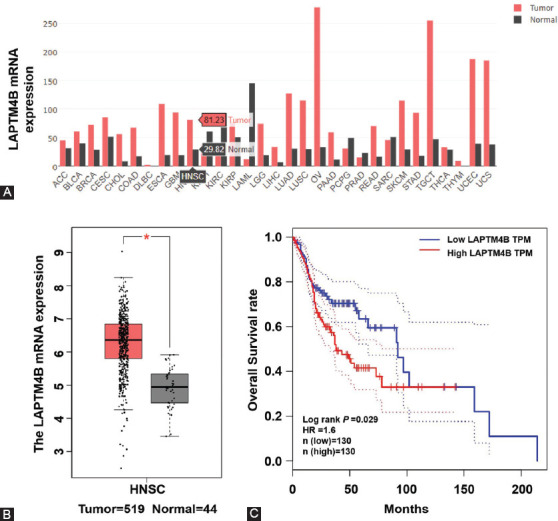
Prediction of LAPTM4B expression in HNSC and its relationship with patient prognosis based on GEPIA online database. (A) *LAPTM4B* was upregulated in most cancer types; (B) the expression level of *LAPTM4B* mRNA in HNSC tissues was significantly higher than that in normal tissues; (C) HNSC patients with high expression of LAPTM4B have worse prognosis. LAPTM4B: Lysosomal protein transmembrane 4 beta; HNSC: Head and neck squamous cell carcinoma; GEPIA: Gene expression profiling interactive analysis; *p < 0.001.

To examine the expression of *LAPTM4B* in human malignant solid tumors, we first utilized data from gene expression profiling interactive analysis (GEPIA; http://gepia.cancer-pku.cn/) for online *in silico* analysis of the available clinical cancer metadata [9]. The results disclosed that *LAPTM4B* was upregulated in multiple cancer samples (Figure 1A). *LAPTM4B* mRNA is upregulated more than 3-fold in head and neck squamous cell carcinoma tissues (HNSC) relative to normal tissues (Figure 1A and B). Meanwhile, the relationship between *LAPTM4B* mRNA expression and the prognosis of HNSC patients was also predicted by GEPIA. The results showed that high *LAPTM4B* expression implies a worse prognosis for HNSC patients (Figure 1C). Thus, the *in silico* predictions suggest that, as the most common NPC tumor subtype, HNSC shows differential expression of *LAPTM4B*.

**FIGURE 2:**
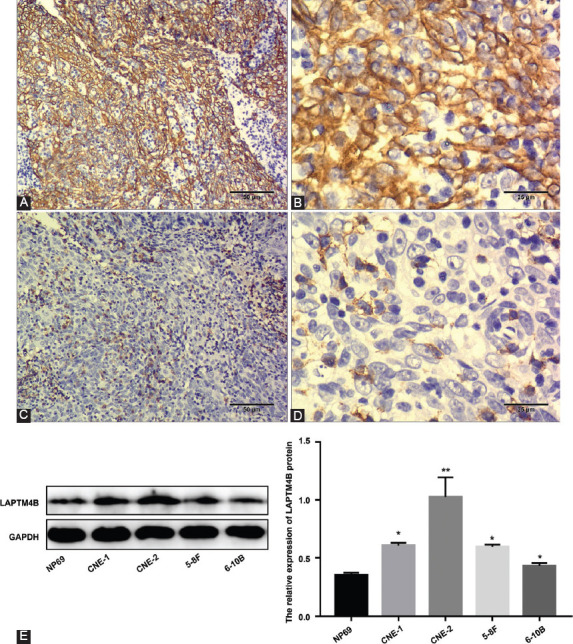
The expression of LAPTM4B protein in NPC. High expression of LAPTM4B protein in NPC tissues (A) ×100 and (B) ×400; low expression of LAPTM4B protein in NPC tissues (C) ×100 and (D) ×400; (E) compared with the immortalized human nasopharyngeal epithelial cell line NP69, LAPTM4B protein was upregulated in NPC cell lines. LAPTM4B: Lysosomal protein transmembrane 4 beta; NPC: Nasopharyngeal carcinoma; *p < 0.05; **p < 0.001.

To explore the relationship between LAPTM4B and NPC, we examined the expression of LAPTM4B protein in NPC tissues by immunohistochemistry. Immunohistochemical assay results suggested that LAPTM4B protein is mainly localized in the cytoplasm and intracellular membranes of NPC cells (Figure 2A-D). At the same time, we examined the expression of LAPTM4B protein in the cell lines by Western blot. As shown in Figure 2E, LAPTM4B was upregulated in NPC cell lines, with higher levels in CNE-1, CNE-2, 5-8F, and 6-10B cells than in NP69, an immortalized human nasopharyngeal epithelial cell line. Among them, the expression of LAPTM4B protein was the highest in CNE-2 cell line. Consequently, we selected the CNE-2 cell line for subsequent functional experiments.

### Higher LAPTM4B expression is associated with poor prognosis of NPC patients

**FIGURE 3:**
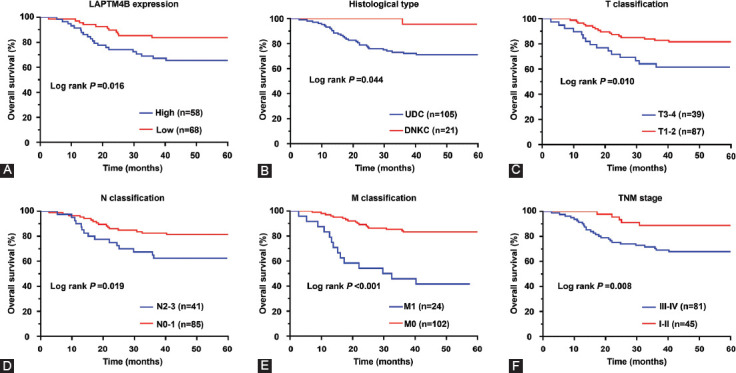
The survival curves for NPC patients. (A) The LAPTM4B expression was associated with poor prognosis of NPC patients; (B) the cancer histological type and prognosis of the respective NPC patients are represented; the T (C), N (D), and M (E) classification and the TNM stage (F) together with the prognosis of the respective NPC patients are also shown. NPC: Nasopharyngeal carcinoma; LAPTM4B: Lysosomal protein transmembrane 4 beta; DNKC: Differentiated non-keratinizing carcinoma; UDC: Undifferentiated carcinoma; TNM: Tumor node metastasis.

Based on the immunohistochemical staining results and evaluation criteria, we divided 126 NPC patients into the low expression (n = 68) and the high expression (n = 58) groups of LAPTM4B. Chi-square test analysis showed that the high expression of LAPTM4B was closely related to the histological subtype of NPC patients and tumor-node-metastasis (TNM) staging (Table 1). Moreover, the survival curves of NPC patients were plotted using OS data. Kaplan–Meier curves indicated that NPC patients in the low LAPTM4B expression group had a better prognosis than those in the high expression group (Figure 3). Given the close relationship between abnormal LAPTM4B expression and the prognosis of NPC patients, a Cox regression model was constructed. Univariate analysis revealed that tissue type, TNM stage, as well as LAPTM4B expression status were associated with OS in NPC patients. Multivariate analysis revealed that high LAPTM4B expression was an independent risk factor predicting poor prognosis in NPC patients (Table 2).

**TABLE 1:**
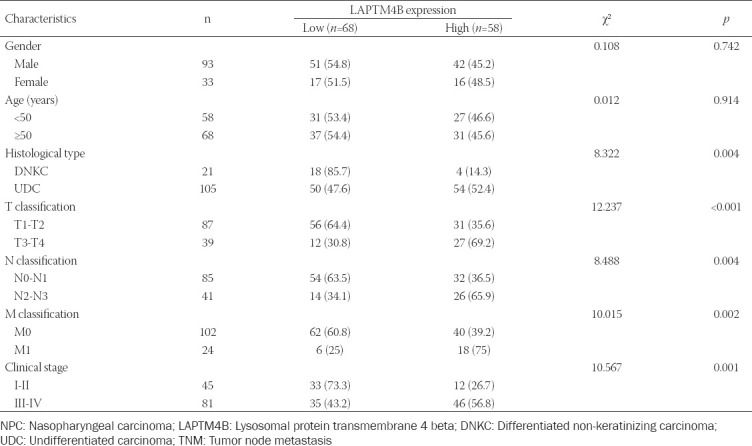
Association between clinicopathological features and LAPTM4B expression in 126 NPC patients

**TABLE 2:**
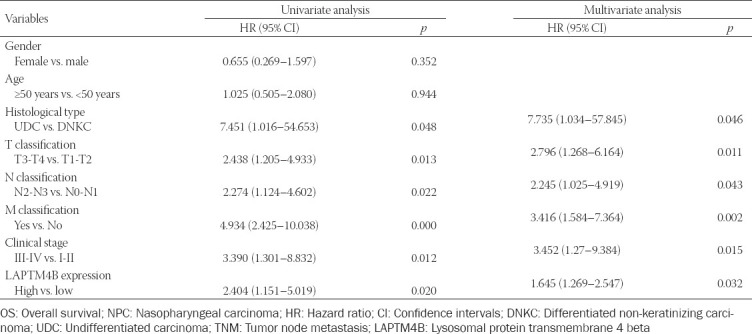
Univariate and multivariate analyses of the OS of 126 patients with NPC

### LAPTM4B promotes proliferation, migration, and invasion of NPC cells

**FIGURE 4:**
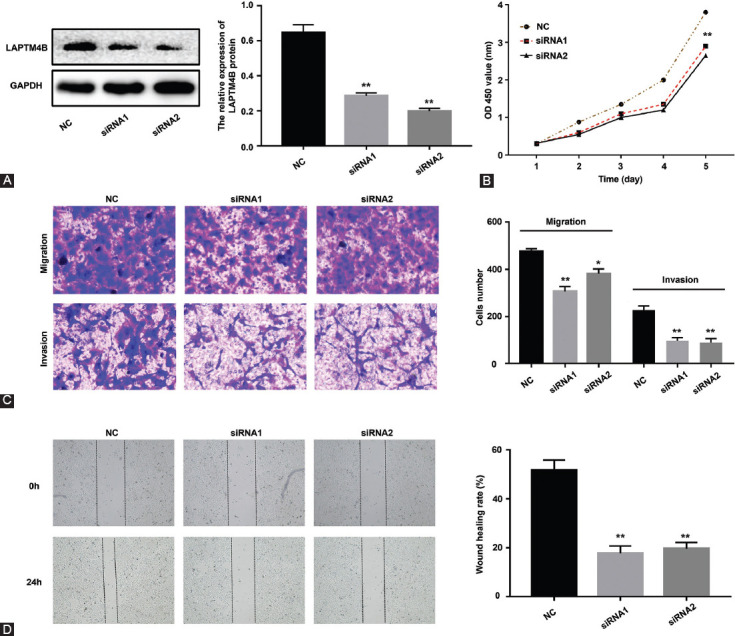
LAPTM4B promotes proliferation, migration, and invasion of NPC cells. (A) LAPTM4B expression was suppressed in CNE-2 cell lines; (B) knockdown of *LAPTM4B* inhibited the proliferation of NPC cells which was assessed by CCK-8 assay; (C) knockdown of *LAPTM4B* inhibited the migration and invasion of NPC cells which was assessed by transwell assay; (D) knockdown of *LAPTM4B* inhibited the migration of NPC cells which was assessed by wound healing assay. LAPTM4B: Lysosomal protein transmembrane 4 beta; NPC: Nasopharyngeal carcinoma; CCK-8: Cell counting kit-8; *p < 0.05; **p < 0.001.

To further examine the role of LAPTM4B in NPC cells, we designed siRNA (siLAPTM4B) to downregulate the endogenous levels of *LAPTM4B* in CNE-2 cells and knockdown efficiency was confirmed by Western blot. Our results revealed that LAPTM4B expression was significantly suppressed by siLAPTM4B in both CNE-2 cell groups as compared with NC group (Figure 4A). Further, we examined the proliferation ability of NPC cells using CCK-8 kit. The results indicated that, compared with the cells in the NC group, siLAPTM4B significantly inhibited the growth of CNE-2 cells on days 4 and 5 (Figure 4B). Next, both wound healing and transwell migration assays were used to observe the effect of LAPTM4B downregulation on NPC cell migration. Matrigel-based transwell assays were used to assess the effect of LAPTM4B depletion on NPC cell invasion. Our data showed that, as compared with the cells in the NC group, the migration of CNE-2 cells (Figure 4C and D) and invasion (Figure 4C) were significantly diminished when they were transfected with siLAPTM4B.

## DISCUSSION

LAPTM4B is an essential membrane protein that contains multiple lysosomal targeting motifs at the C-terminus and colocalizes with endosomal and lysosomal markers in anaphase [10,11]. LAPTM4B is considered a pro-oncogene and can be abnormally upregulated in a variety of malignant solid tumors. Using Northern blot analysis, Shao et al. showed that *LAPTM4B* mRNA expression is upregulated in liver cancer and inversely proportional to the degree of tumor differentiation, but not to the proliferation and survival of tumor cells [12]. In another study, researchers have analyzed the genome-wide expression profiles of solid tumor samples by Affymetrix GeneChip hybridization and found that *LAPTM4B* was significantly upregulated in lung and colorectal cancer samples; Northern blot analysis showed that LAPTM4B was overexpressed in most uterine, breast, and ovarian cancers [13]. In breast cancer, overexpression of LAPTM4B leads to the sequestration of anthracycline, delaying its concentration in the nucleus, thereby inducing anthracycline resistance and ultimately breast cancer recurrence and metastasis [14]. Of note, LAPTM4B was also closely associated with ovarian, cervical, and endometrial cancers [15-17]. In the present study, we show that LAPTM4B protein localizes in the cytoplasm and inner membrane of NPC cells by immunohistochemistry. High expression of LAPTM4B is closely related to multiple clinicopathological features of NPC patients and is an independent risk factor for poor prognosis of NPC patients.

In *in vitro* studies, aberrant expression of LAPTM4B is closely associated with tumor cell growth, proliferation, migration, invasion, apoptosis resistance, autophagy initiation, as well as multidrug resistance [18]. In this study, to explore which cellular processes are regulated by LAPTM4B in NPC, we inhibited the endogenous expression of *LAPTM4B* in NPC cell line CNE-2 by siRNA technology. Subsequent functional evaluations revealed that the knockdown of *LAPTM4B* impedes NPC cell proliferation, migration, and invasion. However, the molecular mechanisms underlying these effects remain unclear. In the available studies, it has been demonstrated that LAPTM4B can promote EGFR signaling in cancer cells and is essential in the process of autophagy triggered by inactive EGFR. Thus, it may serve as a target molecule in cancer therapy [18-20]. Studies have shown that tumor cell proliferation is abrogated by inhibiting LAPTM4B-mediated activation of AKT signaling in addition to disruption of the interaction between LAPTM4B and SH3 domain-containing proteins to control cancer invasion and metastasis [21]. Additionally, inhibition of LAPTM4B reduces the export of late endosomal ceramide, thereby improving anti-apoptotic milieus and allowing LAPTM4B to dissociate from the cell [22].

## CONCLUSION

It is worth noting that this study has certain limitations. For instance, no *in vivo* validation was performed, and an in-depth mechanistic exploration is needed. However, the current study provides important clues regarding the crucial role of LAPTM4B in NPC. To sum up, LAPTM4B plays a cancer-promoting role in the progression of NPC and may be a potential target for NPC therapy.
